# Temporal and Autoregressive Features for Cattle Behavior Classification Using Low-Power LoRaWAN Accelerometer Data

**DOI:** 10.3390/s26123855

**Published:** 2026-06-17

**Authors:** Onur Uysal, Mehmet Emin Bakir, Andres R. Perea, Vedat Tumen, Santiago A. Utsumi

**Affiliations:** 1 Department of Computer Engineering, Izmir Katip Celebi University, 35620 Izmir, Turkey; y230246001@ogr.ikc.edu.tr; 2 Department of Animal and Range Sciences, New Mexico State University, Las Cruces, NM 88003, USA; arperea@nmsu.edu (A.R.P.); sutsumi@nmsu.edu (S.A.U.); 3 Department of Computer Engineering, Bitlis Eren University, 13100 Bitlis, Turkey; vtumen@beu.edu.tr

**Keywords:** cattle behavior classification, precision livestock management, precision agriculture, smart farming, machine learning, time-series analysis, LoRaWAN, accelerometer

## Abstract

Accelerometer sensors and artificial intelligence (AI) are reshaping automated behavior monitoring in precision livestock management, yet their joint deployment on extensive rangelands is constrained by energy and bandwidth budgets. Low-Power Long-Range Wide-Area Network (LoRaWAN) collars address these constraints by compressing the raw tri-axial signal on the device into a single scalar per reporting interval, the Motion Index (MI). This onboard compression preserves enough signal to separate active behaviors but discards the per-axis and frequency content that fine-grained classification typically relies on. On a dataset of 9222 labeled observations from 24 cows across four breeds, MI distinguishes walking from grazing reliably but fails to separate ruminating from resting; both correspond to a stationary animal and yield near-zero, statistically indistinguishable distributions. Earlier MI-only models reached only about 65% four-class accuracy, and ruminating was commonly merged into resting. We show that much of this loss can be recovered by treating the MI stream as a time series. Session-aware lag features, rolling statistics, and an autoregressive previous-behavior feature lift four-class macro-F1 from 0.647 to 0.94, with per-class F1 of 0.95 for ruminating and 0.92 for resting (and at least 0.92 for every behavior). In autonomous deployment the previous behavior must be predicted rather than observed; for this setting we add a Viterbi sequence-decoding step that combines the classifier’s per-step outputs with a learned behavior-transition model, recovering a substantial part of the ruminating signal from the activity stream alone while keeping walking and grazing reliable. The gain is consistent across seven classifiers and four genetically distinct breeds, indicating that it is driven by the features rather than by a specific model.

## 1. Introduction

Sensor technologies and artificial intelligence (AI) are reshaping how agricultural systems are monitored and managed [1,2,3]. In livestock operations, wearable sensors that report animal activity to a back-end analytics pipeline have become a cornerstone of precision livestock management (PLM), enabling automated behavior monitoring at scales that are impractical for direct observation [4]. Behavior budgets derived from these systems provide producers with early indicators of illness, estrus, and forage or nutritional disorders by tracking how animals allocate their time across grazing, walking, and resting.

In extensive arid rangelands, where cattle roam over hundreds to thousands of hectares, energy and bandwidth constraints preclude high-frequency tri-axial streaming, and GPS alone provides location without behavioral context. Low-Power Long-Range Wide-Area Network (LoRaWAN) collars offer a practical alternative: Rather than transmitting the raw signal, the device computes a single scalar, the Motion Index (MI), per reporting interval, and transmits only the summary. In the dataset used here, Abeeway Compact Tracker 2.1.1 devices were fitted to the neck of each cow, configured with an approximately 4 g sensitivity threshold and a 60 s reporting interval [5]. Each sample reaching the server therefore represents hundreds of tri-axial accelerometer readings collapsed into one count.

This compression preserves enough signal to separate active behaviors but obscures finer distinctions among stationary ones. Walking (WA; MI =24.9±3.05) and grazing (GR; MI =13.2±4.23) produce clearly separable MI distributions, while ruminating (RU; MI =1.3±2.29) and resting (RE; MI =2.3±3.21) yield values near zero with no statistically significant pairwise difference (p>0.05, Bonferroni-adjusted) [5]. Consequently, Perea et al. [5] reported a 4-class macro-F1 of only 0.647 when discriminating WA, GR, RU and RE from MI alone, and merged RU into RE to keep the models practically usable.

The per-axis and frequency content discarded on the device cannot be recovered at the server. The contribution of this paper is to show that a large fraction of the resulting loss in discriminative power can nevertheless be compensated for by treating the MI stream as a time series rather than a sequence of independent samples. Working from the publicly available data of Perea et al. [5], comprising 9222 labeled observations from 24 cows across four breeds, we engineer session-aware temporal features that make the short history of each observation explicit, together with an autoregressive feature that encodes the previously labeled behavior (the exact definitions are given in Section 4.4). Evaluated across seven classifiers and across four genetically distinct breeds, the same features lift 4-class macro-F1 from 0.647 to 0.94 and push per-class F1 above 0.92 for every behavior, including RU. The improvement is consistent across classifiers, indicating that it stems from the features rather than from a specific model.

The remainder of the paper is organized as follows. Section 2 reviews related work on cattle behavior classification. Section 3 describes the dataset, sensor system, and study area. Section 4 details the feature engineering and modeling approach. Section 5 presents the experimental results. Section 6 discusses the findings and practical implications. Section 7 concludes.

## 2. Related Work

### 2.1. Precision Livestock Management and Cattle Behavior Monitoring

Automated monitoring of cattle behavior is a central application of precision livestock management (PLM). Bailey et al. [1] highlighted opportunities to apply PLM principles and sensor technologies to optimize livestock production on rangelands. Comprehensive reviews have cataloged the sensing modalities and analytical methods employed for cattle behavior classification in grazing systems [3,4]. Tzanidakis et al. [2] surveyed PLM technologies specifically for grazing animals, noting the growing adoption of wearable accelerometers and GPS-based systems in extensive production environments. Riaboff et al. [6] conducted a systematic review of accelerometer data processing techniques for ruminant behavior prediction, identifying feature extraction strategies, window sizes, and classifier choices as key determinants of classification performance. These reviews consistently highlight the trade-off between sensor data richness and the practical constraints (battery life, communication range, device cost) that govern monitoring in extensive rangeland settings.

### 2.2. Accelerometer-Based Behavior Classification

The majority of wearable cattle behavior classification studies employ tri-axial accelerometers sampling at 1–100 Hz, from which statistical, time-domain, and frequency-domain features are extracted. Seminal work by Martiskainen et al. [7] demonstrated that support vector machines (SVM) applied to tri-axial accelerometer features could classify eight dairy cow behaviors, achieving an overall precision of 78% (κ=0.69) and establishing accelerometers as a viable sensing modality for behavior recognition. Robert et al. [8] evaluated three-dimensional accelerometers for monitoring and classifying cattle behavior patterns, providing early evidence that collar-mounted devices can reliably capture head and neck movement.

Subsequent studies expanded the classifier repertoire and target settings. González et al. [9] applied decision trees to collar-mounted accelerometer and GPS data from grazing steers, classifying five behaviors (foraging, ruminating, resting, traveling, and other active behaviors) with overall accuracies of 85.5% and 90.5% in development and evaluation trials, respectively. Benaissa et al. [10] compared accelerometer placement (leg vs. neck) and SVM-based classifiers for dairy barn behaviors, finding that sensor location strongly influenced classification accuracy. Wang et al. [11] developed an ensemble classifier combining multiple Backpropagation (BP) neural networks with AdaBoost and Dempster–Shafer (D-S) evidence theory for real-time recognition of cow behavior from accelerometer and location data. Sprinkle et al. [12] used random forests on 3-axis accelerometer data to predict grazing, resting, and walking time budgets in rangeland beef cattle, a setting closely related to the present study, and reported prediction errors of 12–14% for grazing and resting time and approximately 30% for walking time when validated against direct observation. Li et al. [13] classified multiple cattle behaviors using *k*-nearest neighbors (KNN), random forest (RF), and XGBoost, reporting that gradient boosting consistently outperformed single classifiers for multi-class problems.

More recently, deep learning approaches have been applied to accelerometer time series. Pavlovic et al. [14] trained convolutional neural networks (CNN) on raw neck-mounted accelerometer signals from cattle, classifying three behaviors (rumination, eating, and other) with an overall F1-score of 0.82 without manual feature engineering. Liu et al. [15] classified cow behavior from IMU signals using a fully convolutional network (FCN), bypassing manual feature extraction while achieving competitive accuracy. Barwick et al. [16], although focused on sheep rather than cattle, demonstrated that tri-axial accelerometers and machine learning (ML) can effectively classify ruminant activity patterns, contributing methodological insights applicable to cattle.

### 2.3. Vision-Based Approaches

An alternative paradigm for cattle behavior monitoring uses computer vision and deep learning on video or image data. Qiao et al. [17] combined 3D convolution with temporal sequence modeling for cow behavior recognition from video, and Gao et al. [18] used a CNN-Bi-LSTM architecture aimed at complex environments with varying lighting and occlusion. Ahmed et al. [19] integrated YOLOv8 with background subtraction to identify stationary and moving cattle from camera imagery, and Daker et al. [20] applied CNN-based classification to visual data. These systems avoid animal-mounted hardware, but they are computationally demanding and constrained by camera coverage, occlusion, and lighting, which limits their use on extensive rangelands where cattle roam over thousands of hectares.

### 2.4. Low-Power LoRaWAN Sensing and the MI Classification Challenge

In extensive arid rangelands, the energy and bandwidth constraints of long-range deployments make high-frequency accelerometer streaming infeasible. LoRaWAN-enabled trackers such as the Abeeway Compact Tracker 2.1.1 address this by computing a single Motion Index (MI) per user-configurable reporting interval and sensitivity, representing a cumulative count of motion events detected by the onboard accelerometer [5]. This extreme data compression, from hundreds of tri-axial samples to one scalar count, enables extended battery life but sacrifices per-axis and frequency-domain features.

Perea et al. [5] evaluated five classical ML models (logistic regression (LR), SVM, multi-layer perceptron (MLP), XGBoost, and RF) on MI-only data from 24 beef cattle instrumented with LoRaWAN collars. All models converged to a macro-F1 of ≈0.927 on 3-class (WA/GR/RE) classification, with no significant inter-model differences. Critically, the authors noted that ruminating (RU) and resting (RE) produced nearly identical MI distributions (mean 1.3±2.29 vs. 2.3±3.21; p>0.05, Bonferroni-adjusted). In preliminary 4-class tests (WA, GR, RU, RE), all classifiers exhibited poor RU classification (macro-F1 = 0.647), with RU instances predominantly misclassified as RE, leading the authors to merge both into a single resting class for their primary analysis. This MI-based classifier has since been integrated into a scalable, end-to-end IoT and remote-sensing platform for operational rangeland and livestock management [21], where it powers a producer-facing behavior-monitoring dashboard. Because the MI-only model cannot reliably separate ruminating from resting, that platform is deployed with the reduced three-class scheme (walking, grazing, and resting), leaving rumination unmonitored in practice. This operational compromise is precisely the four-class bottleneck that the temporal feature framework developed in this work removes.

### 2.5. Research Gap: Temporal Modeling of Low-Resolution MI Data

The studies reviewed above either operate on high-frequency multi-axis accelerometer data [7,9,10,12,14] or on video streams [17,18,19,20], both of which provide rich feature spaces for behavior discrimination. In low-power LoRaWAN deployments, however, the entire input is a single MI scalar per time step, and as Perea et al. [5] demonstrated, this compressed representation cannot distinguish ruminating from resting; the two behaviors produce statistically indistinguishable MI distributions, capping 4-class macro-F1 at 0.647 regardless of the classifier used. Consequently, existing MI-based studies have been limited to 3-class schemes that merge ruminating and resting into a single category, forgoing the finer behavioral resolution needed for applications such as nutritional monitoring and digestive health assessment.

Our work addresses this open problem by introducing a temporal and autoregressive feature engineering framework that exploits the sequential structure of the MI time series. Rather than treating each observation independently, the proposed approach enriches each time step with context from preceding observations, compensating for the information lost during onboard compression. This strategy not only improves 3-class accuracy but, more importantly, enables reliable 4-class classification with high per-class performance for all behaviors, including ruminating, without requiring additional sensors or richer data streams.

## 3. Materials

The dataset, sensor infrastructure, and behavioral annotations used in this study were collected and published by Perea et al. [5]. We reuse their publicly available dataset and reproduce key details of the experimental setup below for completeness; all data collection, animal instrumentation, and behavioral labeling were conducted by Perea et al.

### 3.1. Study Area

Perea et al. [5] conducted the study at two USDA research facilities in the Chihuahuan Desert of southern New Mexico, USA. The Jornada Experimental Range (JER), operated by USDA-ARS, comprised two pastures of 1196 ha and 2235 ha dominated by *Bouteloua eriopoda* (black grama) with scattered *Larrea tridentata* (creosote bush) and *Prosopis glandulosa* (honey mesquite). The Chihuahuan Desert Rangeland Research Center (CDRRC), operated by New Mexico State University, comprised two pastures of 480 ha and 1947 ha with similar vegetation composition.

The region is characterized by an arid climate with mean annual precipitation of approximately 250 mm, concentrated in the July to September monsoon season. Temperatures during the June to November 2022 data collection period ranged from 5 °C to 42 °C.

### 3.2. Animals and Sensor Deployment

Perea et al. [5] instrumented twenty-four mature beef cows (4 to 9 years old) representing four breeds: Angus-Hereford crossbred (AH, n=6), Raramuri Criollo (RC, n=6), Brangus (BR, n=6), and Brahman (BH, n=6).

Each animal was fitted with an Abeeway Compact Tracker 2.1.1 (Abeeway SAS, Biot, France) collar equipped with a tri-axial accelerometer sampling at 12.5 Hz. The device computes a Motion Index (MI), defined as a cumulative count of motion events: a change of one MI unit is registered when the acceleration in any axis exceeds a programmable sensitivity threshold at least three times within a 2 s window. The sensitivity was set to 1% (approximately 4 g), and the reporting interval was configured to 60 s [5]. This configuration was chosen by Perea et al. [5] to balance the detection of behaviorally relevant locomotor activity against battery life and transmission volume: a lower (more sensitive) threshold registers finer movements but increases the motion-event count, packet payload, and power draw, whereas the selected setting reliably captures locomotion while keeping the device within its long-range, low-power energy budget. Because the MI is reported as a cumulative counter (ActivityCount), the per-interval MI is obtained by subtraction of consecutive counter values. In LoRaWAN communication, data packets can be lost; therefore, not all intervals are exactly 60 s. When a packet is missed, the next received packet carries the accumulated count over multiple intervals, resulting in inflated MI values that do not reflect the true per-interval activity rate. Perea et al. [5] addressed missing packets by interpolation using the average MI of the two adjacent received packets, discarding frames with more than five consecutive losses or concurrent behavior changes. The LoRaWAN infrastructure achieved 98.5% packet delivery (1.5% loss) over the study duration [5].

### 3.3. Behavioral Observations and Labeling

Ground-truth behavioral labels were collected by Perea et al. [5] through direct visual observation using GoPro Hero 8 cameras (GoPro, Inc., San Mateo, CA, USA) and trained observers over 170.5 h across the June to November 2022 period. Behaviors were classified into four mutually exclusive categories, following the ethogram defined by Perea et al. [5]:

- Walking (WA): active horizontal displacement with the head up;
- Grazing (GR): biting, chewing, and searching for forage;
- Ruminating (RU): stationary (standing or lying) while chewing cud. Rumination involves small jaw and head movements associated with regurgitation, and cattle typically stretch their necks to facilitate the transit of the bolus from the rumen;
- Resting (RE): stationary (standing or lying), not ruminating and not displacing. This class aggregates all remaining non-locomotor activities, including grooming, scratching, playing, and fly-swatting.

A minimum 30 s duration rule was applied: transient behaviors shorter than 30 s were assigned to the surrounding dominant activity. The resulting dataset comprises 9222 labeled instances with the class distribution in Table 1.

### 3.4. Motion Index Characteristics

Table 2 reproduces the MI descriptive statistics reported by Perea et al. [5]. Using mixed models with cow as a random effect and Bonferroni-corrected pairwise comparisons, Perea et al. [5] reported significant differences (p<0.05) among all behavior pairs except RU vs. RE (p>0.05), assigning both stationary classes to the same post hoc group. Our independent analysis on the same dataset confirms this finding: One-way ANOVA detected significant overall differences (F-statistic =17,961.7, p<0.001), and Tukey Honestly Significant Difference (HSD) test on cow-averaged MI values yielded a borderline RU vs. RE contrast (p=0.047), while Cohen’s effect size [22] d=0.35 indicates a small-to-medium effect with limited practical separability. All other pairwise comparisons produced large effect sizes (d>2.7).

The slightly higher mean MI of RE compared to RU may appear counterintuitive, since rumination involves continuous jaw and head movements while cattle classed as resting can be fully static. We propose, as a post hoc hypothesis, that the sensitivity threshold of approximately 4 g is too coarse to register the small oscillations produced during rumination, whereas the RE class aggregates every non-locomotor, non-rumination activity, including grooming, scratching, playing, and fly-swatting, any of which can briefly exceed the threshold; this interpretation is plausible but was not tested directly. These converging results highlight the challenge motivating this work: MI alone cannot reliably distinguish RU from RE.

**Figure 1:**
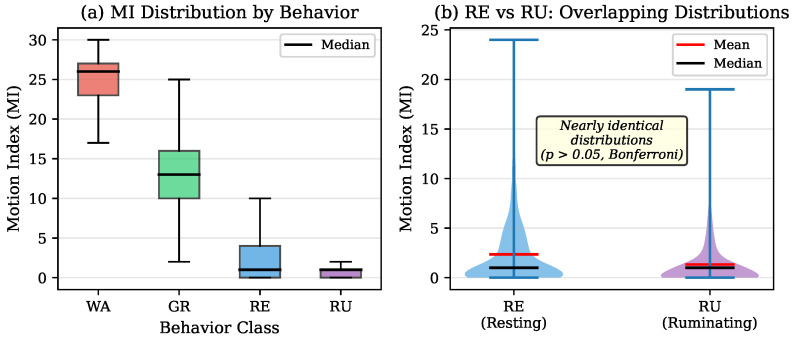
(**a**) Motion Index distributions by behavior class; the black horizontal line inside each box indicates the median. WA and GR are well separated, while RE and RU produce nearly identical distributions concentrated near zero. (**b**) Violin plot comparing RE and RU distributions. Both central tendency measures are nearly identical between classes, highlighting the extensive overlap that prevents discrimination from MI alone.

Figure 1 provides visual evidence of this overlap. While WA and GR produce well-separated MI distributions, RE and RU are concentrated near zero with extensive overlap, confirming that MI alone cannot discriminate these classes.

## 4. Methods

### 4.1. Data Preprocessing

Data were organized as per-animal time series sorted chronologically by the timestamp field. the reporting interval is not always exactly 60 s (due to packet loss or transmission jitter), the time difference Δt between consecutive received packets varies across observations. To account for this variability, we define the Normalized Activity Rate (${\mathrm{NormAct}}_{t}$) as the per-interval Motion Index ${\mathrm{MI}}_{t}$, that is, the number of motion events registered by the onboard accelerometer during the interval ending at time *t*, divided by the elapsed time, Δt, since the previous packet:

(1) $${\mathrm{NormAct}}_{t}=\frac{{\mathrm{MI}}_{t}}{\Delta t}$$

where $\Delta t=t_{\mathrm{current}}-t_{\mathrm{previous}}$ is measured in seconds. This normalization converts the raw cumulative count into an activity rate (motion events per second), making observations with different elapsed intervals comparable. Because NormAct is expressed per second, the variable reporting interval does not require a separate magnitude cut-off: no observation exceeds NormAct=1, and the few long-gap observations (Δt>120 s; 597 in total) are retained, as excluding them changes the four-class macro-F1 by less than 0.01.

The session-aware temporal features defined in Section 4.4 (the two lags, the two rolling means, and the previous-behavior label) require a short but complete history within the same monitoring session (Section 4.2). Consequently, the first one to two observations of every session, together with observations in sessions too short to supply this history, lack a complete feature vector and are excluded from modeling. This requirement reduces the 9222 labeled observations to 7657 model-ready observations; the resulting class distribution is given in Table 3. Ruminating, the rarest class, accounts for 725 of these observations (9.5%), compared with 10.6% of the original 9222 (Table 1).

### 4.2. Session Detection

Temporal feature engineering requires that observations within each computational window belong to the same continuous monitoring bout. Large temporal gaps between consecutive observations (caused by device sleep, packet loss, or management handling) would introduce spurious lag and rolling-mean values if treated as contiguous.

To address this, we partition each animal’s time series into *observation sessions* by scanning for temporal discontinuities exceeding a threshold of τ=900 s (15 min). A new session begins whenever Δt>τ, and all autoregressive features are computed strictly within session boundaries, with NaN values assigned at the first observation(s) of each session where insufficient history exists. This procedure yielded 324 sessions across the 24 animals (mean 13.5±4.2 per cow), with a median session length of 13 observations and a median session duration of approximately 19 min. The 900 s threshold was adopted as a robust default rather than a tuned value. Varying it from 5 to 60 min (Table 4) lowers the number of detected sessions smoothly as the threshold grows, while the four-class macro-F1 stays between 0.941 and 0.946. Performance therefore does not depend on the specific value of τ, and 900 s sits well inside this stable range.

Session-aware feature engineering is critical to prevent information leakage from temporally disjoint monitoring bouts.

### 4.3. Evidence for Temporal Patterns

Before constructing temporal features, we investigated whether cattle behaviors exhibit sufficient temporal structure to justify an autoregressive approach.

**Figure 2:**
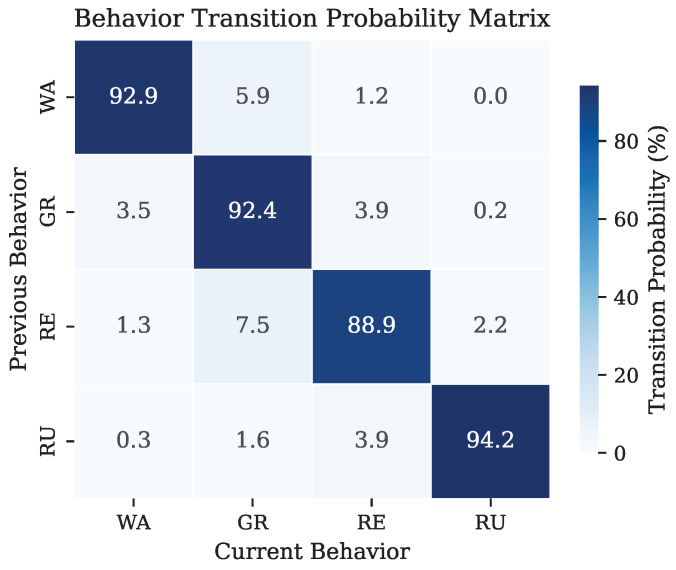
Behavior transition probability matrix computed from within-session consecutive observation pairs (n=7333 transitions). Each row represents the behavior at time t−1, and each column represents the behavior at time *t*; cell values give $P(y_{t}|y_{t-1})$ as percentages. Diagonal values indicate strong behavioral persistence (88.9 to 94.2%); off-diagonal values reveal distinct transition patterns between RU and RE despite their similar MI distributions.

**Transition probabilities.** We computed the empirical transition probability matrix $P(y_{t}|y_{t-1})$ across all within-session consecutive observation pairs (n=7333 transitions). In this matrix (Figure 2), each row represents the behavior at time t−1 (previous observation), and each column represents the behavior at time *t* (current observation); thus, each cell gives the conditional probability that an animal exhibits a given behavior at time *t*, given its behavior at time t−1. All four behaviors exhibit strong self-transition (persistence) probabilities along the diagonal: WA → WA = 92.9%, GR → GR = 92.4%, RU → RU = 94.2%, and RE → RE = 88.9%. These high diagonal values indicate that cattle behaviors are temporally persistent. Once an animal begins a behavior, it is very likely to remain in that state at the next observation.

Critically, the off-diagonal elements reveal that RU and RE, despite producing nearly identical MI values, exhibit distinct transition patterns. When an animal is ruminating, it very rarely transitions directly to WA (0.3%) or GR (1.6%), consistent with the sustained posture associated with cud chewing. By contrast, resting animals transition to GR considerably more often (7.5%), and less frequently transition to RU (2.2%) and WA (1.3%), reflecting a greater overall tendency to change state. RU shows correspondingly low outgoing transitions to RE (3.9%), WA (0.3%), and GR (1.6%). These asymmetric off-diagonal patterns confirm that RU and RE, while producing similar MI values, occupy distinct positions in the behavioral sequence and are distinguishable through their temporal context.

**Figure 3:**
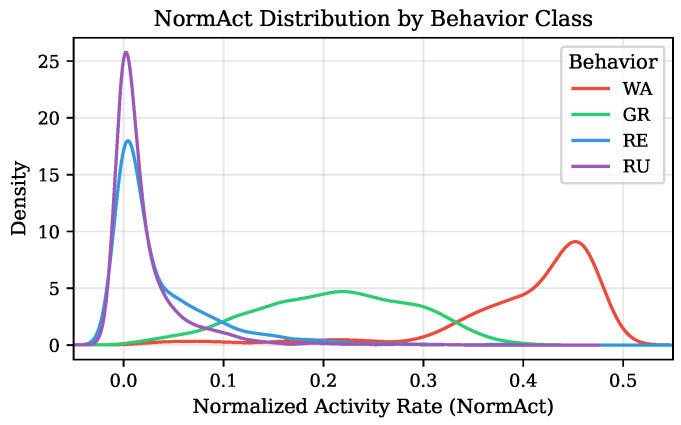
Kernel density estimates of Normalized Activity Rate (NormAct) by behavior class. RE and RU produce heavily overlapping distributions near zero, confirming the insufficiency of the instantaneous activity rate for discriminating these classes.

**NormAct distributions.** Figure 3 shows the kernel density estimates of NormAct by behavior class. While WA (mean NormAct = 0.40) and GR (mean = 0.21) are well separated, RE (mean = 0.04) and RU (mean = 0.02) produce heavily overlapping distributions concentrated near zero. This overlap confirms that instantaneous NormAct alone cannot discriminate these classes. However, the strong but distinct temporal persistence patterns (Figure 2) suggest that the sequence of NormAct values and preceding behaviors can provide the discriminative signal that instantaneous values lack.

### 4.4. Temporal and Autoregressive Feature Engineering

Based on the evidence of strong temporal structure, we construct the following temporal features for each observation at time *t*, computed strictly within each animal’s observation sessions. These comprise time-series features derived from past NormAct values (lag and rolling statistics) and one autoregressive label-feedback feature (previous activity encoding):

1. Lag-1 (NormAct_Lag1): $x_{t-1}$, the NormAct value at the previous time step.
2. Lag-2 (NormAct_Lag2): $x_{t-2}$, the NormAct value two steps prior.
3. Rolling Mean-3 (Roll_Mean_3): ${\bar{x}}_{t,3}=\frac{1}{3}\sum_{i=1}^{3}x_{t-i}$, the moving average over the 3 most recent *past* observations (excluding $x_{t}$).
4. Rolling Mean-5 (Roll_Mean_5): ${\bar{x}}_{t,5}=\frac{1}{5}\sum_{i=1}^{5}x_{t-i}$, the moving average over the 5 most recent past observations.
5. Previous Activity Encoding (Prev_Activity_Enc): $y_{t-1}\in \left\{0,1,\ldots ,K-1\right\}$, the integer-encoded behavioral label at the previous time step, where *K* is the number of classes (3 or 4).

Observations lacking sufficient history (i.e., at session boundaries) were assigned NaN values and excluded from training. The final feature vector is
  (2) $$x_{t}=\left[{\mathrm{NormAct}}_{t},\;x_{t-1},\;x_{t-2},\;{\bar{x}}_{t,3},\;{\bar{x}}_{t,5},\;y_{t-1}\right]$$

These six features capture three complementary aspects of temporal context: instantaneous activity (NormAct), recent activity trajectory (the lag and rolling-window features, which capture whether the animal is accelerating, decelerating, or maintaining a sustained activity level), and behavioral inertia via the autoregressive label-feedback feature Prev_Activity_Enc, which exploits the strong self-transition probabilities shown in Figure 2.

### 4.5. Classification Model

Two classification problems were considered:

- 3-class: Walking (WA), Grazing (GR), and Resting (RE, with RU merged into RE);
- 4-class: Walking (WA), Grazing (GR), Resting (RE), and Ruminating (RU).

Both formulations follow the ethogram of Perea et al. [5], who evaluated the 4-class problem but achieved only a macro-F1 of 0.647 due to poor RU/RE separability, and therefore focused their primary analysis on the 3-class setting.

We evaluated seven classifiers on both problems using the same temporal feature set and 5-fold stratified cross-validation (CV), a protocol in which the data are split into five folds of roughly equal size, each fold taking a turn as the test set while the remaining four are used for training, and performance is averaged across the five rounds. The seven classifiers comprised the five used by Perea et al. [5] (logistic regression (LR), support vector machine with radial basis function kernel (SVM-RBF), multi-layer perceptron (MLP), random forest (RF), and XGBoost [23]) together with two additional gradient boosting frameworks, LightGBM [24] and CatBoost [25].

CatBoost achieved the highest cross-validated macro-F1 on both the 3-class (0.953) and 4-class (0.943) problems, and was therefore selected as the primary model for subsequent analysis. Beyond its empirical ranking, CatBoost is particularly suited for the proposed feature set, as it natively handles the categorical Prev_Activity_Enc feature without one-hot encoding, its ordered boosting scheme reduces overfitting on sequentially correlated data, and it provides built-in feature importance analysis.

Hyperparameters of the selected CatBoost model were then optimized via grid search with 3-fold cross-validation on the training set. The search space comprised tree depth ∈{4,6,8}, number of iterations ∈{150,200,300}, and learning rate ∈{0.05,0.1,0.15} (27 combinations). Optimal parameters were: depth =6, iterations =200, learning rate =0.05 for the 3-class problem; and depth =6, iterations =300, learning rate =0.1 for the 4-class problem.

### 4.6. Evaluation Protocol

To ensure robust and comprehensive evaluation, we employed five complementary assessment strategies, each addressing a distinct aspect of model validity:

1. 5-fold stratified cross-validation (model comparison): All five classifiers used by Perea et al. [5] (LR, SVM, MLP, RF, XGBoost) plus CatBoost and LightGBM were compared using the same temporal feature set under identical 5-fold CV, verifying that performance gains stem from the features rather than the choice of classifier.
2. Hold-out test set: A 70/30 stratified random split, maintaining class proportions. The training set (n=5359) was used for model training and hyperparameter tuning; the test set (n=2298) was reserved for final evaluation, providing per-class confusion matrices and detailed error analysis that are difficult to aggregate across CV folds.
3. 10-fold stratified cross-validation: The full dataset was partitioned into 10 stratified folds, yielding overall macro-F1 and accuracy with 95% confidence intervals. As this protocol uses all data for both training and testing (in rotation), it provides tighter and less partition-dependent performance estimates than a single hold-out split.
4. Bootstrap confidence intervals: In each of 100 iterations, 80% of the data was sampled with replacement to train a fresh CatBoost model, and the remaining out-of-bag observations served as the test set. Per-class precision, recall, and F1 were recorded for every iteration, and the 2.5th and 97.5th percentiles of the resulting 100-value distributions define the 95% confidence intervals. Given that each iteration sees a different random subset of the data, the width of the interval reflects how sensitive each metric is to the particular observations included; narrow intervals indicate stable, reproducible performance. This complements the 10-fold CV, which reports aggregate-level CIs, by providing uncertainty estimates at the individual-class level, critical for substantiating the claim that all classes, including the challenging RU class, achieve F1 ≥0.92.
5. Leave-One-Breed-Out cross-validation (LOBO-CV): Each of the four breeds was held out in turn while the model was trained on the remaining three breeds. This protocol evaluates whether the learned temporal patterns generalize across genetically distinct cattle populations, a question that neither random-split CV nor the hold-out test can answer because both mix breeds across training and test partitions.

### 4.7. Deployable Inference Without Observed Labels

The previous-behavior feature uses the true label of the preceding observation. This is the appropriate setting for the cross-validation above, which measures how well the behaviors separate once that context is given, and for retrospective analysis of recorded data. In fully autonomous online use, the previous label is not observed and has to be replaced by a prediction. We therefore evaluate three inference regimes, all under leave-animals-out cross-validation with disjoint training and test animals so that the cost of running without the observed label is separated from the effect of the data split.

1. Teacher-forced (upper bound). The full model with the true previous label, as in the main results.
2. Closed-loop autoregressive inference. Each session is processed in time order, and the model’s own prediction at step t−1 is fed back as the previous-behavior input at step *t*. The first *K* observations of each session are produced by a model that does not use the previous label (a NormAct-only warm-up), after which the autoregressive model takes over.
3. Sequence decoding. Instead of feeding hard predicted labels back one step at a time, we treat each session as a hidden Markov sequence. A model trained without the previous-label feature supplies per-class emission probabilities from the NormAct features, the transition matrix $P(y_{t}|y_{t-1})$ is estimated on the training sessions, and the most likely behavior sequence is recovered by Viterbi decoding [26,27]. Since maximum-likelihood decoding tends to under-recover rare states, we add a single class-prior offset for the minority ruminating class and report it as an operating curve that trades ruminating recall against resting precision, rather than as a tuned constant.

For comparison we also report a temporal-only model that omits the previous-behavior feature and uses only the leakage-free NormAct lag and rolling features so that it can be deployed directly without any label feedback.

## 5. Results

### 5.1. Model Comparison

We evaluated all five models used by Perea et al. [5] (LR, SVM, MLP, RF, XGBoost) alongside two additional gradient boosting frameworks (LightGBM, CatBoost). All classifiers were trained on the same temporal feature set in order to determine whether the observed improvements are attributable to the features or to a particular classifier. Table 5 reports 5-fold stratified cross-validation macro-F1 scores and hold-out test macro-F1 for each model.

Several observations are noteworthy. First, all seven classifiers substantially outperform the MI-only baseline: even the simplest model (logistic regression) achieves a 4-class test macro-F1 of 0.913, an improvement of 0.266 over Perea et al.’s 0.647. This confirms that the performance gains are driven by the temporal features, not the choice of classifier. Second, the top five models span three different model families (gradient boosting, neural network, kernel-based) yet all achieve 4-class macro-F1 ≥0.935, with only 0.007 separating the best (MLP, 0.942) from the fifth-ranked (Random Forest, 0.935). Third, CatBoost achieves the highest 3-class CV macro-F1 (0.953) and the highest 4-class CV macro-F1 (0.943), while MLP marginally edges it on 4-class test F1 (0.942 vs. 0.941); we retain CatBoost as the primary model for subsequent analyses owing to its best cross-validated performance. This 0.001 gap is negligible and, if anything, reinforces the central point that performance is set by the temporal features rather than by a particular classifier. Crucially, Perea et al.’s own RF and XGBoost, which converged to 0.927 macro-F1 (3-class) and 0.647 (4-class) using MI alone, now achieve macro-F1 of 0.935 and 0.938 (4-class) with temporal features, demonstrating that the same classifiers perform dramatically better when given temporally enriched inputs.

### 5.2. Tuned CatBoost Performance

We conducted hyperparameter tuning on the best-performing model, CatBoost. Table 6 summarizes the performance of the tuned model. On the 3-class problem (WA, GR, RE, with RU merged into RE following Perea et al. [5]), the model achieved a hold-out test macro-F1 of 0.95 on the 70/30 stratified split ($n_{\mathrm{test}}=2298$) and a 10-fold cross-validated macro-F1 of 0.95 (95% CI: [0.95, 0.96]). Perea et al. reported a macro-F1 of 0.927 on the same 3-class problem using MI alone with five classical ML models.

On the 4-class problem, which includes the previously problematic RU class, the model achieved a test macro-F1 of 0.94 and a cross-validated macro-F1 of 0.94 (95% CI: [0.94, 0.95]). Perea et al. [5] reported that 4-class classification using MI alone yielded a macro-F1 of only 0.647, with RU instances predominantly misclassified as RE. Our temporal features raise the 4-class macro-F1 from 0.647 to 0.94, a 0.29 absolute improvement. The modest macro-F1 drop of only 0.01 between our 3-class and 4-class settings demonstrates that RU/RE discrimination is achievable with minimal sacrifice in overall performance.

Table 7 provides a direct comparison between the baseline MI-only approach of Perea et al. [5] and our temporal feature framework.

### 5.3. Per-Class Analysis

Table 8 reports per-class precision, recall, and F1-score for the 4-class tuned model, with 95% bootstrap confidence intervals on F1-score. All four classes achieve F1-scores ≥ 0.92. Notably, RU attains an F1-score of 0.95 (95% CI: [0.93, 0.96]) despite being the rarest class (9.5% of the dataset) and having MI distributions statistically indistinguishable from RE. The high RU precision (0.98) indicates that when the model predicts RU, it is almost always correct; the slightly lower recall (0.92) reflects the difficulty of catching all RU instances given their MI overlap with RE. Across all four classes, the bootstrap confidence intervals are narrow (spanning 0.02 to 0.03), indicating that the reported per-class performance is stable across resampled test sets and is not an artifact of a particular data split.

### 5.4. Confusion Matrix Analysis

**Figure 4:**
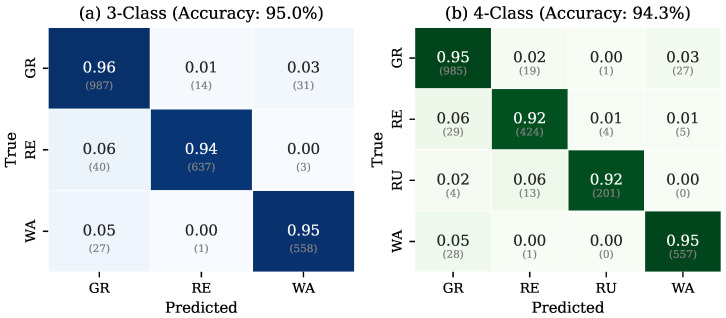
Normalized confusion matrices for (**a**) 3-class and (**b**) 4-class classification. Cell values show proportions (with raw counts in parentheses). In the 4-class setting, 92.2% of RU instances are correctly classified despite MI overlap with RE.

Figure 4 presents the normalized confusion matrices for both classification problems. In the 3-class setting (Figure 4a), all classes achieve recall ≥0.94, with RE (which includes merged RU) reaching 0.94 recall. In the 4-class setting (Figure 4b), the primary source of error is the RU↔RE confusion: 13 of 218 RU instances (6.0%) are misclassified as RE, and 4 RU instances (1.8%) as GR. Conversely, only 4 of 462 RE instances (0.9%) are misclassified as RU. This asymmetric error pattern reflects the inherent difficulty: during rumination, the gentle jaw and head movements may produce MI values indistinguishable from those of complete rest, causing the model to default to the more prevalent RE class. The temporal features suppress most of this confusion by using behavioral persistence and transition context. The residual confusion is concentrated at the boundaries of rumination bouts rather than spread evenly across them. When ruminating is analyzed by bout, the first observation of a bout is recovered only about 47% of the time and is otherwise most often labeled RE, whereas later observations within an established bout are classified almost perfectly (recall above 0.99), and short bouts of one to three observations are harder than longer ones. The errors therefore correspond to brief rumination episodes and to the onset minute of a new bout, where the recent activity history still reflects the preceding behavior and the low-amplitude movements of rumination remain close to rest at the device’s sensitivity.

### 5.5. Feature Importance

**Figure 5:**
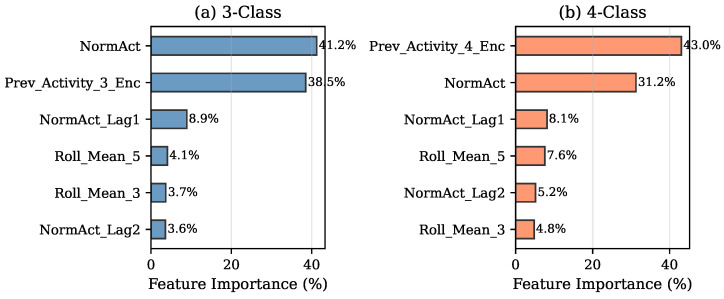
CatBoost feature importance for (**a**) 3-class and (**b**) 4-class models. In the 4-class setting, Prev_Activity_Enc becomes the dominant feature, reflecting the model’s increased reliance on behavioral sequence information to discriminate RU from RE.

Figure 5 presents the CatBoost feature importance scores from the tuned models. In the 3-class setting, NormAct (41.2%) is the dominant feature, followed closely by Prev_Activity_Enc (38.5%). In the 4-class setting, their roles reverse: Prev_Activity_Enc becomes the most important feature (43.0%), with NormAct contributing 31.2%. This shift is highly informative: When the model must distinguish RU from RE (behaviors with nearly identical NormAct values), it relies more heavily on knowing *what the animal was doing previously*, exploiting the distinct transition probability patterns shown in Figure 2. The remaining temporal features (NormAct_Lag1, NormAct_Lag2, Roll_Mean_3, Roll_Mean_5) collectively contribute 20.3% (3-class) and 25.7% (4-class), capturing recent activity trajectory information.

The same ranking holds for the other classifiers. Table 9 reports the 4-class importances for CatBoost, XGBoost, and Random Forest. All three rank the previous-behavior feature first and NormAct second, and a model-agnostic permutation-importance check gives the same two features as the most important for every model. This agreement supports the conclusion that the gain is driven by the features rather than by a particular classifier.

### 5.6. Feature Ablation and Deployable Inference

Two questions follow from the dominance of the previous-behavior feature: how much of the gain it contributes on its own, and how the model behaves when the true previous label is not available, as in autonomous deployment. We examine both below. An ablation isolates the contribution of each feature group, and a set of deployment experiments replaces the true previous label by a prediction.

To separate the contributions, Table 10 reports 4-class performance on the model-ready set under stratified 5-fold cross-validation for three feature sets. NormAct alone reproduces the MI-only regime (macro-F1 0.63, with ruminating essentially unrecovered). Adding the leakage-free temporal features (the two lags and two rolling means) raises macro-F1 to 0.71 and gives the first non-trivial separation of ruminating (RU F1 0.29). Adding the previous-behavior label lifts macro-F1 to 0.94 and RU F1 to 0.95. The temporal NormAct features thus provide a deployable improvement on their own, and most of the remaining gain comes from the previous-behavior label.

As the previous label is replaced by the model’s own prediction in deployment, Table 11 compares the three inference regimes of Section 4.7 under leave-animals-out cross-validation. Feeding predictions back does not reproduce the teacher-forced result. With no warm-up the ruminating class collapses (RU F1 0), and with a short NormAct-only warm-up the macro-F1 settles near 0.68 with RU F1 near 0.24. That is below the temporal-only model (macro-F1 0.69), because the autoregressive model was trained to trust a previous label that is now noisy, and an error at a bout onset carries into the next step. Lengthening the warm-up beyond about five observations does not help. Walking and grazing stay reliable throughout (F1 about 0.90 or higher), so the loss is confined to the ruminating and resting substates.

Treating each session as a hidden Markov sequence and decoding it globally avoids committing to a single hard previous label at each step. With the empirical transition matrix and a modest prior offset for the rare ruminating class, Viterbi decoding raises the 4-class macro-F1 to 0.71 and recovers ruminating recall to about 0.47 with no ground-truth labels at inference; a higher ruminating prior pushes recall up to about 0.83 at the cost of resting precision, so the prior acts as an operating point set by the desired ruminating recall. Sequence decoding is therefore the preferred fully autonomous option when ruminating recall matters, while the teacher-forced model remains appropriate for retrospective analysis of recorded data and for deployments that periodically re-anchor the previous-behavior input through direct observation.

### 5.7. Leave-One-Breed-Out Validation

**Figure 6:**
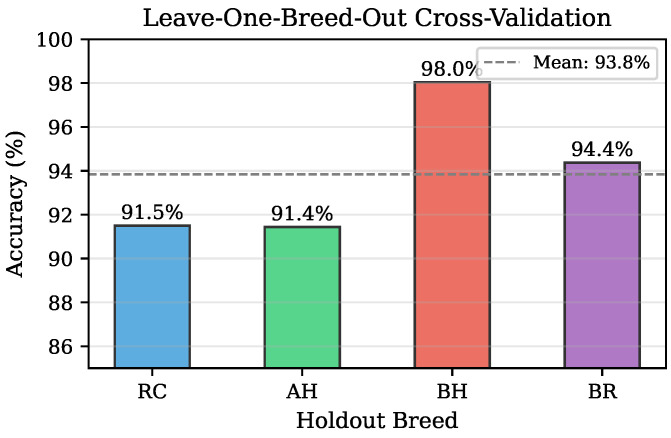
Leave-One-Breed-Out cross-validation accuracy by holdout breed. The dashed line indicates the mean accuracy (93.8%) across all four breed holdouts. All breeds exceed 91% accuracy, demonstrating robust cross-breed generalization.

Leave-One-Breed-Out cross-validation (LOBO-CV) assessed whether the temporal behavioral patterns generalize across genetically distinct cattle populations. Table 12 and Figure 6 present the results. The mean accuracy across breed holdouts was 93.84% (±3.12%), with a mean macro-F1 of 0.94, and all individual breeds exceeding 91% accuracy.

These results indicate that the model generalizes effectively across breeds: Even when an entire breed is excluded from training, the temporal features learned from the remaining three breeds are sufficient to classify the held-out breed’s behaviors with high accuracy. This is a practically important finding, as it suggests the model can be deployed on breeds not represented in the training data without substantial performance degradation. Brahman (BH) yielded the highest accuracy (98.05%), indicating that this breed’s behavioral patterns are well captured by the temporal dynamics of the other three breeds. Raramuri Criollo (RC) and Angus–Hereford (AH) yielded the lowest accuracies (91.50% and 91.44%, respectively), which may reflect breed-specific behavioral strategies. For instance, Raramuri Criollo cattle are adapted to arid environments and are known to exhibit different foraging and movement patterns [32]. Nevertheless, even these lowest-performing breeds achieved accuracies above 91%, confirming that the fundamental temporal structure of cattle behavior (high behavioral persistence and distinct transition patterns) is conserved across genetically diverse populations and that the proposed approach is robust to breed variation.

## 6. Discussion

### 6.1. Why Temporal Context Succeeds Where MI Alone Fails

The central finding of this work is that temporal and autoregressive features enable fine-grained cattle behavior classification from a single-scalar MI time series, a data source previously considered too impoverished to distinguish between ruminating and resting substates. The mechanism underlying this improvement can be understood through the transition probability analysis (Figure 2).

Although RU and RE produce nearly identical instantaneous MI values (means 1.3 and 2.3, respectively; p>0.05), they exhibit distinct temporal signatures. RU shows the highest self-transition probability (94.2%) and minimal transitions to active behaviors (RU → WA: 0.3%, RU → GR: 1.6%), consistent with the sustained stationary posture associated with cud chewing: Once a cow begins ruminating, it tends to remain in that state for extended periods. RE shows a lower self-transition probability (88.9%) and higher total outgoing transition rates (RE → GR: 7.5%, RE → RU: 2.2%, RE → WA: 1.3%), reflecting the greater readiness of a resting animal to change state.

The autoregressive features capture these patterns in three complementary ways. First, the Prev_Activity_Enc feature directly encodes behavioral inertia: If the previous observation was RU, the strong self-transition probability makes it likely that the current observation is also RU. Second, the lag and rolling-mean features capture the sustained low-activity trajectory characteristic of rumination bouts, as opposed to the more variable activity patterns of resting (which is more likely to be preceded or followed by brief movement). Third, the combination of all six features provides a multi-scale temporal context that the model can use to disambiguate otherwise identical instantaneous signals.

The feature importance analysis (Figure 5) confirms this interpretation: When moving from 3-class to 4-class classification, the relative importance of Prev_Activity_Enc increases from 38.5% to 43.0%, while NormAct decreases from 41.2% to 31.2%. This shift demonstrates that the model adaptively relies more on temporal context precisely when it faces the RU/RE discrimination task.

### 6.2. Comparison with Prior Work

Table 13 places our results in the context of existing cattle behavior classification studies, reporting macro-F1 where available and overall accuracy otherwise to facilitate comparison across studies that differ in their reported metrics.

Direct comparison across studies is complicated by differences in sensor richness, number of behaviors, reported metrics, and, critically, sample size. Studies with few animals (e.g., Li et al. with 10 cattle) may report higher performance partly because limited inter-individual variability makes the classification task easier; models trained and tested on a small, homogeneous cohort are less likely to encounter the behavioral diversity present in larger herds. Conversely, larger studies face greater inter-individual variability: Sprinkle et al. used 48 cows over a 2-year trial, reporting prediction errors of 12–30% depending on behavior class, and Martiskainen et al. used 30 cows and 8 behavior classes, the most ambitious classification scheme in this comparison, and accordingly reported lower per-class performance.

Among all studies listed, only Perea et al. [5] and the present work share the same dataset, sensor, animals, and labeling protocol, making this the only truly one-to-one comparison. On this common ground, our temporal feature engineering raises the 4-class macro-F1 from 0.647 to 0.94, a 0.29 absolute improvement, and improves 3-class macro-F1 from 0.927 to 0.95.

A further distinguishing aspect of our approach is the use of temporal and autoregressive features. None of the other studies in Table 13 exploit inter-observation temporal dependencies: Each employs windowed statistical or frequency-domain features computed independently within fixed time segments, without modeling transitions between consecutive behavioral states. Our framework introduces lag features, rolling statistics, and previous-activity encoding that capture behavioral persistence and transition dynamics, which proved essential for discriminating the otherwise indistinguishable RU and RE classes.

The extended model comparison (Table 5) provides strong evidence that the improvement is feature-driven rather than model-driven. When Perea et al.’s own classifiers (RF and XGBoost) are retrained with temporal features on the same dataset, their 4-class macro-F1 jumps from 0.647 to 0.935 and 0.938, respectively, a controlled comparison in which only the feature set changes. Moreover, even logistic regression, the simplest model tested, achieves a 4-class macro-F1 of 0.91, vastly exceeding the MI-only baseline. These results suggest that temporal context, rather than multi-axis high-frequency sensor data or more sophisticated models, is sufficient for fine-grained behavior discrimination from low-power livestock monitoring systems, an important consideration for large-scale rangeland deployments where battery life and infrastructure cost are critical constraints.

### 6.3. Breed Generalization

The LOBO-CV results (Table 12) demonstrate that the temporal behavioral patterns exploited by our model are not breed-specific artifacts. Performance varied across breeds, with Brahman yielding the highest accuracy (98.05%, macro-F1 = 0.98) and Raramuri Criollo and Angus-Hereford the lowest (91.50% and 91.44%, respectively; macro-F1 = 0.92 for both). While the source of this inter-breed variation is beyond the scope of the present study, the consistently high performance across all four genetically distinct populations (mean accuracy 93.84%, mean macro-F1 = 0.94, all breeds >91%) confirms that the fundamental temporal structure of cattle behavior, namely high behavioral persistence and distinct transition patterns, generalizes across breeds. This is a practically important result: The model can be deployed on breeds not represented in the training data without substantial performance degradation.

### 6.4. Practical Considerations

The previous-behavior feature is the single largest contributor to the 4-class result, so its behavior in deployment deserves direct treatment. During training, and in the cross-validation above, the true previous label is used; in fully autonomous operation only a predicted label is available, which raises the question of error propagation. Section 5.6 answers this empirically rather than by argument. Feeding predicted labels back in a closed loop does not preserve the teacher-forced performance: The 4-class macro-F1 falls to about 0.68 and ruminating F1 to about 0.24, below even a model that omits the previous-label feature (macro-F1 0.69). The error analysis of Section 5.4 explains why: Ruminating errors occur mainly at bout onsets, and a wrong onset prediction is then carried forward as the previous-behavior input. Lengthening the NormAct-only warm-up helps only marginally and plateaus by about five observations.

Three deployment regimes follow from this. (i) Where recorded sequences are analyzed after the fact, or where the previous-behavior input is re-anchored periodically by direct observation, the full model applies and the 0.94 result holds. (ii) For fully autonomous operation, the temporal NormAct features without the previous label give reliable walking and grazing estimates (F1 near 0.92) at an overall macro-F1 of 0.69, and should be preferred over closed-loop feedback. (iii) When ruminating recall is the priority, sequence decoding with a transition model recovers ruminating recall to between about 0.47 and 0.83 with no ground-truth labels at inference, at a cost in resting precision that the user controls through the ruminating prior. In all three regimes the walking and grazing budgets stay reliable, and the ruminating and resting substates are where performance depends on how the previous-behavior context is supplied.

### 6.5. Limitations

Several limitations should be considered when interpreting these results. First, the dataset comprises 7657 usable observations from 24 animals. While the four-breed, two-site design enhances generalizability, larger multi-site datasets would strengthen claims of broad applicability. Second, all data originate from a single sensor type (Abeeway Compact Tracker 2.1.1) in Chihuahuan Desert rangelands; performance on different sensor platforms or in different ecosystems remains to be validated. Third, class imbalance (RU accounts for only 9.5% of observations) may affect reliability of RU-specific metrics, although the bootstrap confidence intervals provide uncertainty quantification. Fourth, the session detection threshold (τ=900 s) was set heuristically, although the sensitivity analysis in Section 4.2 shows that performance is stable across thresholds from 5 to 60 min. Finally, although the 10-fold CV and LOBO-CV protocols provide robust population-level estimates, leave-one-animal-out cross-validation would provide stronger guarantees of individual-level generalization.

## 7. Conclusions

This work shows that a large part of the information lost when a tri-axial accelerometer signal is compressed onboard to a single Motion Index can be recovered at the analysis stage by exploiting the temporal structure that survives the compression. Session-aware lag features, rolling statistics, and an autoregressive previous-behavior encoding turn the MI stream into a representation rich enough to separate ruminating from resting, the two behaviors that MI alone cannot distinguish. Detailed performance figures, per-class confidence intervals, and the cross-classifier and cross-breed results that support this claim are reported in Section 5 and Section 6.

The key insight is that, although RU and RE produce nearly identical instantaneous MI values, they exhibit distinct temporal signatures: different self-transition probabilities, different transition patterns to neighboring behaviors, and different activity trajectories. The temporal and autoregressive features capture these signatures, enabling discrimination that is out of reach for static features alone. The gain is consistent across seven classifiers and across four genetically distinct breeds, indicating that it is driven by the features rather than by a specific model or by breed-specific artefacts.

The practical implication is that fine-grained behavior monitoring need not require richer sensors or higher bandwidth. Producers deploying low-power LoRaWAN collars on extensive rangelands can obtain rumination and resting time budgets, which are established indicators of animal health, welfare, and nutritional status, from the same compressed stream that is already being transmitted.

For deployment we suggest the following. Use the temporal NormAct features, or sequence decoding when ruminating recall is the priority, for autonomous online classification, and reserve the full previous-label model for retrospective analysis or for settings where the previous behavior can be re-anchored by occasional direct observation. Closed-loop label feedback is not recommended. Retraining or recalibration is warranted whenever the sensor configuration, reporting interval, collar placement, breed composition, or season and forage conditions change, and otherwise on a seasonal schedule as new labeled observations become available.

Validation on additional sensor platforms, ecosystems, and larger multi-site herds would further establish the generalizability of the approach.

## Figures and Tables

**Table 1:** Class distribution in the cattle behavior dataset.

Behavior	Code	Count	Proportion (%)
Walking	WA	2286	24.8
Grazing	GR	3928	42.6
Ruminating	RU	976	10.6
Resting	RE	2032	22.0
Total		9222	100.0

**Table 2:** Motion Index statistics by behavioral class, reproduced from Perea et al. [5]. Post hoc groups are based on Bonferroni-adjusted pairwise comparisons; behaviors sharing the same letter are not significantly different (p>0.05).

Behavior	Mean MI	SD	Post-Hoc Group
Walking (WA)	24.9	3.05	a
Grazing (GR)	13.2	4.23	b
Ruminating (RU)	1.3	2.29	c
Resting (RE)	2.3	3.21	c

**Table 3:** Class distribution of the 7657 model-ready observations, after session-aware feature construction.

Behavior	Code	Count	Proportion (%)
Walking	WA	1952	25.5
Grazing	GR	3440	44.9
Ruminating	RU	725	9.5
Resting	RE	1540	20.1
Total		7657	100.0

**Table 4:** Effect of the session-gap threshold τ on the number of detected sessions, the size of the model-ready set, and four-class performance (CatBoost, 5-fold cross-validated macro-F1).

τ (min)	Sessions	Model-Ready Obs.	4-Class Macro-F1
5	559	7160	0.946
10	375	7547	0.946
15	324	7657	0.943
20	294	7731	0.943
30	265	7788	0.942
60	214	7923	0.941

**Table 5:** Extended model comparison using the temporal feature set. All seven classifiers, including the five used by Perea et al. [5] with MI alone, were trained on the same temporal features. Macro-F1 is reported for 5-fold CV and hold-out test set. CatBoost is listed first as the primary model; remaining rows are sorted by 4-Cl. test macro-F1. Cl. = Class. The best result per column is shown in bold.

Model	3-Cl. CV F1	3-Cl. Test F1	4-Cl. CV F1	4-Cl. Test F1
CatBoost [25]	0.953	0.951	0.943	0.941
MLP [28]	0.949	0.948	0.939	0.942
XGBoost [23]	0.951	0.947	0.941	0.938
SVM (RBF) [29]	0.942	0.941	0.934	0.936
Random Forest [30]	0.951	0.945	0.937	0.935
LightGBM [24]	0.948	0.944	0.936	0.929
Logistic Reg. [31]	0.930	0.925	0.915	0.913
Perea et al. [5] MI-only baseline (best of five models):				
SVM/MLP/XG/RF	n/a	0.927 ^a^	n/a	0.647

^a^ Macro-F1 from Perea et al. [5], LR scored 0.906; the four other models converged to 0.927.

**Table 6:** Tuned CatBoost performance on 3-class and 4-class classification. Hold-out test metrics and 10-fold stratified cross-validation results with 95% confidence intervals.

Problem	Test Acc.	Test Macro-F1	CV Acc. [95% CI]	CV Macro-F1 [95% CI]
3-Class	0.95	0.95	0.95 [0.95, 0.96]	0.95 [0.95, 0.96]
4-Class	0.94	0.94	0.95 [0.94, 0.95]	0.94 [0.94, 0.95]

**Table 7:** Direct comparison between the MI-only baseline (Perea et al. [5], best-performing model) and our temporal feature approach (tuned CatBoost). Both approaches were evaluated on the same dataset of 9222 labeled observations from 24 cattle.

Metric	Perea et al. [5]	This Work
3-Class Macro-F1	0.927	0.95 [0.95, 0.96]
4-Class Macro-F1	0.647	0.94 [0.94, 0.95]
Feature Set	MI only (1 feature)	NormAct + 5 temporal (6 features)

**Table 8:** Per-class metrics for the 4-class tuned CatBoost model. Precision and recall are point estimates from the hold-out test set; F1-score 95% confidence intervals are from 100-iteration bootstrap resampling.

Class	Support	Precision	Recall	F1-Score [95% CI]
GR (Grazing)	1032	0.94	0.95	0.95 [0.94, 0.96]
RE (Resting)	462	0.93	0.92	0.92 [0.91, 0.93]
RU (Ruminating)	218	0.98	0.92	0.95 [0.93, 0.96]
WA (Walking)	586	0.95	0.95	0.95 [0.94, 0.96]

**Table 9:** Relative feature importance (%) for the 4-class problem across three classifiers (gain-based importance, computed on the same 70/30 split). The previous-behavior feature ranks first and NormAct second for all three.

Model	Prev_Act.	NormAct	Lag1	Lag2	Roll_3	Roll_5
CatBoost	43.0	31.2	8.1	5.2	4.8	7.6
XGBoost	82.4	8.9	1.3	1.0	4.5	1.9
Random Forest	41.0	24.5	8.8	2.5	11.6	11.5

**Table 10:** Feature ablation for 4-class classification (stratified 5-fold cross-validation, CatBoost). The previous-behavior feature uses the true previous label. Per-class values are F1-scores.

Feature Set	Macro-F1	GR	RE	RU	WA
NormAct only	0.63	0.90	0.71	0.01	0.90
NormAct + lags and rolling means	0.71	0.92	0.69	0.29	0.93
Full model (with previous label)	0.94	0.95	0.92	0.95	0.95

**Table 11:** Deployable inference under leave-animals-out cross-validation (4-class). The teacher-forced row uses the true previous label and is the upper bound; every other row uses no ground-truth label at inference. *K* is the NormAct-only warm-up length, in observations. Per-class values are F1-scores; RU rec. is ruminating recall.

Inference Regime	Macro-F1	GR	RE	RU	WA	RU Rec.
Teacher-forced (true previous label)	0.94	0.94	0.92	0.95	0.94	0.94
Temporal-only (no previous label)	0.69	0.91	0.68	0.26	0.92	0.19
Closed-loop, no warm-up	0.58	0.84	0.69	0.00	0.80	0.00
Closed-loop, warm-up K=1	0.67	0.89	0.67	0.22	0.90	0.15
Closed-loop, warm-up K=5	0.68	0.90	0.70	0.24	0.90	0.16
Closed-loop, warm-up K=10	0.68	0.90	0.69	0.24	0.91	0.16
Sequence decoding (Viterbi)	0.71	0.91	0.58	0.43	0.92	0.47

**Table 12:** Leave-One-Breed-Out cross-validation results for 4-class classification. Each breed was held out as the test set while the remaining three breeds served as training data.

Holdout Breed	*n* (Test)	Accuracy (%)	Macro-F1
Raramuri Criollo (RC)	2189	91.50	0.92
Angus–Hereford (AH)	1986	91.44	0.92
Brahman (BH)	1688	98.05	0.98
Brangus (BR)	1794	94.37	0.94
Mean ± SD		93.84 ± 3.12	0.94 ± 0.03

**Table 13:** Comparison with selected cattle behavior classification studies. #Cl. = number of behavioral classes; *n* = number of animals. The performance column reports macro-F1 where available and overall accuracy otherwise, as indicated by the Metric column. Since prior studies report heterogeneous evaluation metrics, direct numerical comparison should be interpreted cautiously.

Study	Sensor	*n*	#Cl.	Perf. (%)	Metric	Model
Martiskainen et al. [7]	Tri-axial accel.	30	8	78 ^a^	Prec.	SVM
González et al. [9]	Accel. + GPS	58	5	86 to 91	Acc.	Decision tree
Sprinkle et al. [12]	Tri-axial accel.	48	3	12 to 30 ^b^	Err.	Random forest
Li et al. [13]	6-axis IMU	10	6	94 ^c^	F1	XGBoost
Perea et al. [5]	LoRaWAN MI	24	3	92.7	F1	RF/XGBoost
Perea et al. [5]	LoRaWAN MI	24	4	64.7 ^d^	F1	RF/XGBoost
This work	LoRaWAN MI	24	3	95	F1	CatBoost
This work	LoRaWAN MI	24	4	94	F1	CatBoost

^a^ Overall precision; the paper reports per-class sensitivities of 65–80% for major behaviors and κ=0.69 but does not provide a single overall accuracy or F1 figure. ^b^ Percentage error between accelerometer-predicted and observed daily behavior time budgets (2017 data); lower values indicate better prediction. The study reports error rather than accuracy. ^c^ Average F1 score at the 60 s window; the study classified six unitary behaviors using a neck-mounted IMU. ^d^ Macro-averaged F1.

## Data Availability

The cattle behavior dataset used in this study was provided by Perea et al. [5] and is available upon request from the original authors.

## Abbreviations

The following abbreviations are used in this manuscript:

AI	Artificial Intelligence
CV	Cross-Validation
GR	Grazing
LOBO	Leave-One-Breed-Out
LoRaWAN	Long Range Wide Area Network
LR	Logistic Regression
MI	Motion Index
ML	Machine Learning
MLP	Multi-Layer Perceptron
PLM	Precision Livestock Management
RE	Resting
RF	Random Forest
RU	Ruminating
SVM	Support Vector Machine
WA	Walking
JER	Jornada Experimental Range
CDRRC	Chihuahuan Desert Rangeland Research Center
